# Dendritic potassium channel dysfunction may contribute to dendrite degeneration in spinocerebellar ataxia type 1

**DOI:** 10.1371/journal.pone.0198040

**Published:** 2018-05-30

**Authors:** Ravi Chopra, David D. Bushart, Vikram G. Shakkottai

**Affiliations:** 1 Medical Scientist Training Program, University of Michigan Medical School, Ann Arbor, Michigan, United States of America; 2 Neuroscience Graduate Program, University of Michigan, Ann Arbor, Michigan, United States of America; 3 Department of Molecular and Integrative Physiology, University of Michigan, Ann Arbor, Michigan, United States of America; 4 Department of Neurology, University of Michigan Medical School, Ann Arbor, Michigan, United States of America; National Institutes of Health, UNITED STATES

## Abstract

Purkinje neuron dendritic degeneration precedes cell loss in cerebellar ataxia, but the basis for dendritic vulnerability in ataxia remains poorly understood. Recent work has suggested that potassium (K^+^) channel dysfunction and consequent spiking abnormalities contribute to Purkinje neuron degeneration, but little attention has been paid to how K^+^ channel dysfunction impacts dendritic excitability and the role this may play in the degenerative process. We examined the relationship between K^+^ channel dysfunction, dendritic excitability and dendritic degeneration in spinocerebellar ataxia type 1 (SCA1). Examination of published RNA sequencing data from SCA1 mice revealed reduced expression of several K^+^ channels that are important regulators of excitability in Purkinje neuron dendrites. Patch clamp recordings in Purkinje neurons from SCA1 mice identified increased dendritic excitability in the form of enhanced back-propagation of action potentials and an increased propensity to produce dendritic calcium spikes. Dendritic excitability could be rescued by restoring expression of large-conductance calcium-activated potassium (BK) channels and activating other K^+^ channels with baclofen. Importantly, this treatment combination improves motor performance and mitigates dendritic degeneration in SCA1 mice. These results suggest that reduced expression of K^+^ channels results in persistently increased dendritic excitability at all stages of disease in SCA1, which in turn may contribute to the dendritic degeneration that precedes cell loss.

## Introduction

Neuronal loss in the cerebellum and its associated pathways is a consistent feature of degenerative cerebellar ataxia [1, 2]. In particular, cerebellar Purkinje neurons, which provide the sole output of the cerebellar cortex, are often prominently involved. Human autopsy studies using samples from patients with cerebellar ataxia reveal atrophy of the normally extensive dendritic arbor in surviving Purkinje neurons, suggesting a neuropathological progression which begins with dendritic degeneration and progresses to cell death [2–4]. Progressive changes in Purkinje neuron morphology are also present in mouse models of cerebellar ataxia, where Purkinje neuron dendritic degeneration consistently precedes detectable cell loss. Motor impairment in these models typically occurs shortly before or coincident with dendritic degeneration, suggesting that dendritic degeneration may contribute to motor impairment [5–7]. Despite the fact that these findings point to dendritic degeneration as an early and clinically relevant process in ataxia neuropathology, the mechanisms underlying dendritic degeneration remain poorly understood.

Cerebellar Purkinje neurons are able to support autonomous spiking in the absence of synaptic input, a property which depends crucially on appropriate function of a number of potassium (K^+^) channels [8–10]. Much is known about how perturbations in K^+^ channels affect Purkinje neuron spiking [11–13], including a number of studies which have identified changes in K^+^ channels that produce aberrant spiking in mouse models of cerebellar ataxia [14–17]. Notably, targeting K^+^ channels involved in aberrant spiking slows Purkinje neuron degeneration in several ataxia models [15, 18, 19]. The majority of the Purkinje neuron membrane is in the dendritic arbor, and many of the K^+^ channels which have been linked to disease are highly expressed and functionally important in the dendritic membrane [20–22]. This raises the possibility that abnormal dendritic physiology secondary to K^+^ channel dysfunction may be a meaningful feature of disease. It is therefore important to explore changes in K^+^ channel function and their impact on dendritic membrane excitability in disease, as well as to explore the relationship between altered dendritic excitability and dendrite loss.

In this study, we utilize a model of spinocerebellar ataxia type 1 (SCA1) where K^+^ channel dysfunction and spiking abnormalities have been linked to neurodegeneration [15, 18], and we explore the hypothesis that reduced expression of K^+^ channels found in Purkinje neuron dendrites results in increased dendritic excitability that then contributes to dendritic degeneration. Examination of published RNA sequencing data from this SCA1 model reveals that among all dysregulated voltage-gated ion channels, there are many downregulated K^+^ channel genes, a number of which are expressed in Purkinje neuron dendrites. Whole-cell patch clamp recordings reveal increases in dendritic excitability in association with this reduced expression, resulting in increased back-propagation of action potentials and a reduced threshold for eliciting dendritic calcium spikes. We demonstrate that a treatment strategy which targets K^+^ channels is able to reduce Purkinje neuron dendritic excitability, and we also find that this treatment strategy improves motor performance and preserves dendrite structure in SCA1 mice. These findings suggest a possible role for dendritic membrane excitability in Purkinje neuron dendrite pathology observed in cerebellar ataxia.

## Materials and methods

### Mice

The current study was approved by the University of Michigan Institutional Animal Care and Use Committee (IACUC). Research was performed in accordance with the U.S. Government Principles for the Utilization and Care of Vertebrate Animals Used in Testing, Research, and Training. Mice were housed in microisolator cages with a maximum of five animals per cage and provided with food and water *ad libitum*. Mice were singly housed only if there was fighting, injuries or due to attrition. Enrichment was provided to all mice. This was in the form of at least one kind nesting material (nestlet, envirodry or an enviropak) and a secondary enrichment option (a tube). ATXN1[82Q] transgenic mice [23] overexpress mutant human ATXN1 with 82 CAG repeats selectively in cerebellar Purkinje neurons under the Purkinje neuron-specific murine *Pcp2 (L7*) promoter and were maintained on an FVB/NJ background (Jackson Labs). ATXN1[82Q] mice were maintained homozygous for the transgene [15]. Age- and sex-matched wild-type FVB/NJ mice were used as controls. A total of 206 mice were used for the experiments. The number of mice of each group for individual experiments is noted either in the legend to each figure or the figure charts. Mice were euthanized at specific time points following isoflurane anesthesia for electrophysiology studies, and histologic analysis. Intracerebellar injection of AAV was performed under continuous isoflurane anesthesia, and following analgesia prior to surgery with carprofen. Mice were monitored continuously for at least an hour following the surgery. Mice were placed on a warming pad and monitored following the surgery for signs of dehydration. Physiologic solution was administered IP/SC as needed to maintain hydration. On the day of surgery, mice were monitored every 4 hours for 8 hours and subsequently monitored daily. Analgesia with carprofen (5 mg/kg) was administered preemptively and also daily for seven days. Humane end points were used, and if there were signs of infection, cerebral hemorrhage, paralysis, or inability to feed, mice would have been euthanized through carbon dioxide overdose followed by decapitation. No animals were euthanized for signs of distress. For all drug studies, mice were weighed weekly. If mice began to lose weight, or there was evidence of poor grooming, or other signs of distress, the trial with that dose of compound would have been terminated and animals would have been euthanized. No animals exhibited signs of distress in this study.

### Analysis of K^+^ channel gene expression changes in RNA sequencing data

**Fig 1:**
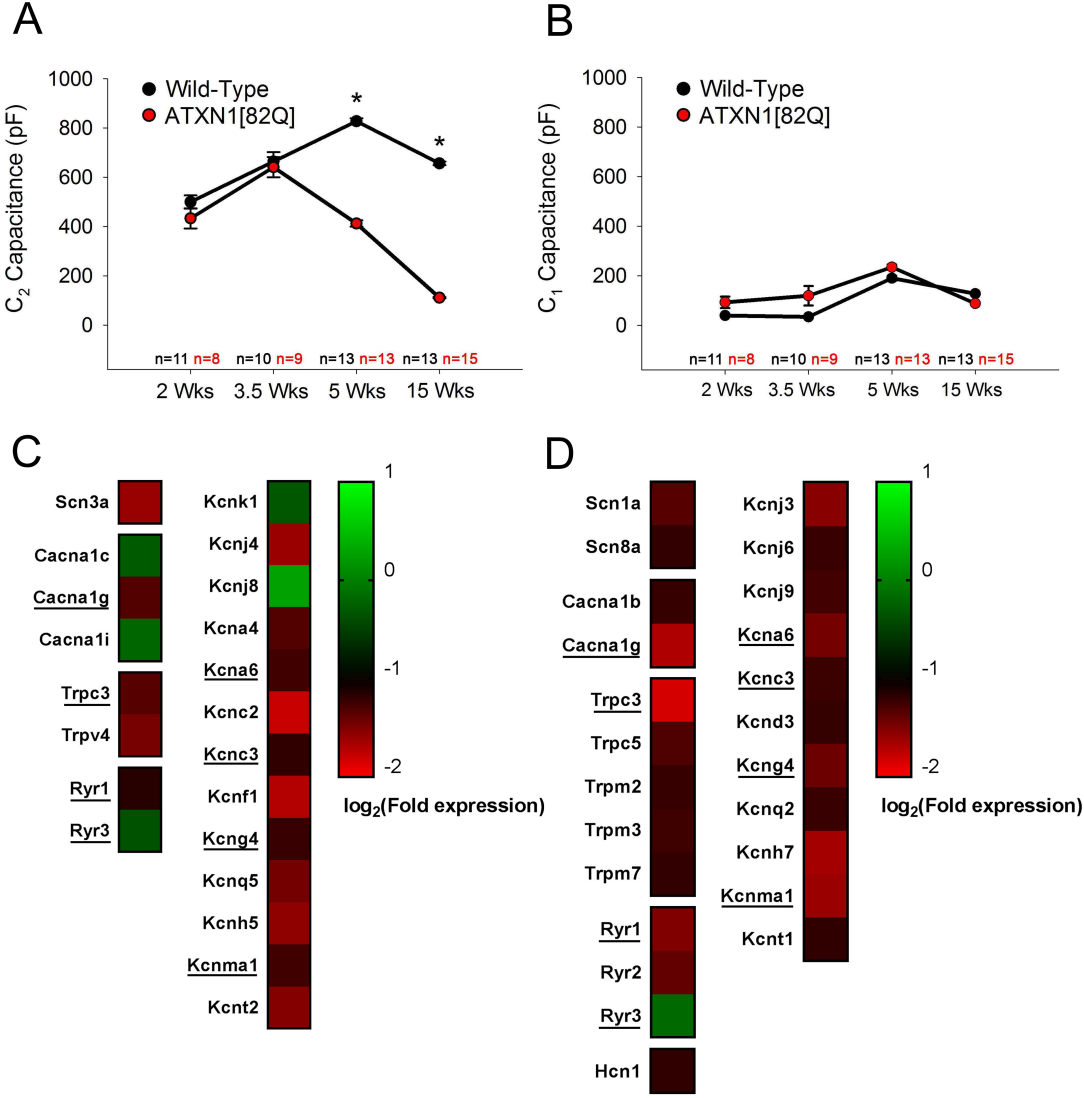
Dendritic degeneration in SCA1 is associated with reduced expression of K^+^ channels found in Purkinje neuron dendrites. **(A)** Measurement of the capacitance of the distal dendritic arbor (C_2_) reveals dendritic degeneration starting at five weeks of age in ATXN1[82Q] Purkinje neurons. **(B)** Measurement of the capacitance of the soma and proximal dendrite (C_1_) reveals no change in ATXN1[82Q] Purkinje neurons. **(C and D)** Evaluation of published RNA sequencing data [24] for all voltage-gated ion channel genes reveals altered expression of several subfamilies of ion channel genes in ATXN1[82Q] mice at five weeks of age **(C)** and twelve weeks of age **(D)**. Only ion channel genes where a statistically significant change in expression at these ages is shown here. The entire list of all known 145 ion channel genes with official IUPHAR classification [25] whose expression was assessed is provided in S1 and S2 Tables. K^+^ channel genes make up a significant proportion of dysregulated genes at both ages. Those genes where expression is dysregulated at both five and twelve weeks of age are underlined. In **(A-B)** N = 3–6 mice were utilized per group. Where error bars are present, data are mean ± SEM. * = *P<0*.*05*. Statistical significance derived by two-way ANOVA **(A**, **B)**.

Published data tables from a previous RNA sequencing analysis of gene expression in ATXN1[82Q] mice [24] were downloaded from the NCBI Gene Expression Omnibus (accession number: GSE75778). Data tables comparing ATXN1[82Q] cerebellar samples to FVB wild-type samples were selected, and the fold change expression (ATXN1[82Q] relative to wild-type), p-value, and q-value for all 145 voltage-gated ion channel genes with official IUPHAR classification [25] were extracted from these data tables (summarized in S1 and S2 Tables). Those ion channel genes where differential expression showed q≤0.05 were defined as statistically-significantly altered in their expression, consistent with the original analysis [24]. Heat maps presented in Fig 1C and 1D reflect log_2_ conversion of the fold change expression for ATXN1[82Q] samples compared to wild-type samples, as obtained from these data tables.

### Electrophysiology

#### Solutions

Artificial CSF (aCSF) contained the following (in mM): 125 NaCl, 3.5 KCl, 26 NaHCO_3_, 1.25 NaH_2_PO_4,_ 2 CaCl_2_, 1 MgCl_2_, and 10 glucose. For all recordings except for measurements of capacitance, pipettes were filled with internal recording solution containing the following (in mm): 119 K Gluconate, 2 Na gluconate, 6 NaCl, 2 MgCl_2_, 0.9 EGTA, 10 HEPES, 14 Tris-phosphocreatine, 4 MgATP, 0.3 tris-GTP, pH 7.3, osmolarity 290 mOsm (somatic recordings) or 285 mOsm (dendritic recordings). For measurements of capacitance, pipettes were filled with internal recording solution containing the following (in mM): 140 CsCl, 2 MgCl_2_, 1 CaCl_2_, 10 EGTA, 10 HEPES, 4 Na_2_ATP, pH 7.3, osmolarity 287 mOsm.

#### Preparation of brain slices for electrophysiological recordings

Mice were anesthetized by isoflurane inhalation, decapitated, and the brains were submerged in pre-warmed (34°C) aCSF. Slices were prepared in aCSF held at 34–36°C on a VT1200 vibratome (Leica) while being continuously bubbled with carbogen (95% 0_2_/5% CO_2_). Slices were prepared to a thickness of 300 μm for somatic recordings and a thickness of 250 μm for dendritic whole-cell recordings. Although ice-cold cutting solution has been used by us previously for preparing cerebellar slices for Purkinje neuron recordings [15,17], we discovered that sectioning at 34°C better preserved dendritic architecture, and the number of viable Purkinje neurons. Once slices were obtained, they were incubated in continuously carbogen-bubbled aCSF for 45 minutes at 34°C. Slices were subsequently stored in continuously carbon-bubbled aCSF at room temperature until use.

#### Patch clamp recordings

Purkinje neurons from cerebellar lobules II-V were identified for patch clamp recordings in parasagittal cerebellar slices using a 40x water-immersion objective and Eclipse FN1 upright microscope (Nikon) with infrared differential interference contrast (IR-DIC) optics that were visualized using NIS Elements image analysis software (Nikon). Borosilicate glass patch pipettes were pulled with resistances of 3–5 MΩ for somatic recordings or 7–10 MΩ for dendritic whole-cell recordings. Recordings were made 1–5 hours after slice preparation in a recording chamber that was continuously perfused with carbogen-bubbled ACSF at 33°C at a flow rate of 2–3 mls/min. Data were acquired using an Axon CV-7B headstage amplifier, Axon Multiclamp 700B amplifier, Digidata 1440A interface, and pClamp-10 software (MDS Analytical Technologies). In all cases, acquired data were digitized at 100 kHz. Voltage data were acquired in current-clamp mode with bridge balance compensation and filtered at 2 kHz. Cells were rejected if the series resistance changed by >20% during recording. Whole-cell somatic recordings were rejected if the series resistance rose above 15 MΩ, with the majority of recordings having a series resistance of <10 MΩ. Whole-cell dendritic recordings were rejected if the series resistance fell outside a range of 15–30 MΩ. All voltages are corrected for a liquid junction potential of +10 mV [26].

Acquisition of data for compartment-specific capacitance measurements was performed in the presence of 50 μM picrotoxin. Capacitative transients were obtained in voltage-clamp mode using 1 second steps to -90 mV from a holding potential of -80 mV. Recordings were excluded if the measured input resistance was <100 MΩ.

Recordings to determine the current threshold for eliciting dendritic calcium spikes were performed as described previously, but in aCSF containing 1 μM tetrodotoxin (Tocris Bioscience). In experiments where baclofen was used, baclofen (Cat. no. B5399, Sigma-Aldrich) was used at a concentration of 2 μM.

#### Analysis of cell capacitance

Determination of dendritic capacitance was performed using a well-established method for analysis of a two-compartment equivalent circuit representing the Purkinje neuron [27]. Briefly, 1 second voltage steps from -80 mV to -90 mv were performed ten times and the recorded currents from each recording were averaged. The average was low-pass filtered at 5 kHz, and input resistance was corrected offline, at which point the decay of the capacitative transient was fit using a two-exponential decay function described below (Eq 1):

$$I(t)=A_{1}e^{-\frac{t}{\tau_{1}}}+A_{2}e^{-\frac{t}{\tau_{2}}}$$ (1)

The constants from each cell’s decay function were then be used to obtain four parameters: C_1_ (representing the capacitance of the soma and main proximal dendrites, Eq 2), C_2_ (representing the capacitance of the distal dendritic arbor, Eq 3), R_1_ (representing the pipette access resistance and internal resistance of the soma and proximal dendrite, Eq 4), and R_2_ (representing the composite internal resistance of dendritic segments separating the distal dendritic arbor from the main proximal dendritic segments, Eq 5). This was done as follows (equations described previously [27]):

$$C_{1}=\frac{{\tau_{1}(A_{1}+A_{2})}^{2}}{A_{1}\Delta V}$$ (2)

$$C_{2}=\frac{A_{2}\tau_{2}}{\Delta V}$$ (3)

$$R_{1}=\frac{\Delta V}{A_{1}+A_{2}}$$ (4)

$$R_{2}=\frac{\Delta V}{A_{2}}-\frac{\Delta V}{A_{1}+A_{2}}$$ (5)

In our measurements of C_1_ and C_2_, the absolute value of C_1_ and C_2_ are reported in Fig 1B and 1A, respectively. Two-way ANOVA revealed that R_access_ was not statistically influenced by age (P = 0.8647) or genotype (P = 0.7688), and there was no apparent interaction (P = 0.1642).

#### Analysis of dendritic whole-cell patch clamp recordings

Analysis of back-propagating action potential amplitude was performed on recordings that are low-pass filtered at 5 kHz. Back-propagating action potential amplitude was defined as the difference between the peak and anti-peak membrane potentials of each spike across a single 10 second trace obtained during dendritic whole-cell patch clamp recording, with the mean of all such measurements reported as the back-propagating action potential amplitude for each data point. Somatic peak-to-trough action potential amplitude was determined by subtracting the peak and anti-peak membrane potentials of each spike across a single 10 second trace obtained during somatic whole-cell patch clamp recordings, with the mean of all such measurements reported as the peak-to-trough action potential amplitude for each cell.

Analysis of action potential half-width in somatic and dendritic recordings was performed on five consecutive spikes from a recording initiated one minute after achieving the whole-cell configuration and low-pass filtered at 5 kHz, with presented data for each cell representing the mean of those five spikes. Half-width was defined as the spike width at half the distance between action potential threshold (defined as 5% of the maximal dV/dt) and action potential peak.

Soma-to-patch distance was determined using still IR-DIC images captured during recording, and measurement was done using shortest-distance free-form line measurements which originate at the center of an ellipse that just encloses the soma and end at the position where the patch pipette is in contact with the dendritic membrane. Measurements were performed in NIS Elements (Nikon), and the experimenter was blind to the genotype of the visualized neuron during measurement.

#### Analysis of dendritic calcium spike threshold

At five weeks of age, the majority of ATXN1[82Q] Purkinje neurons have undergone depolarization block, and their membrane potential can be restored using 1 μM tetrodotoxin and 100 μM CdCl_2_ [15]. We similarly observed non-firing cells in five week old ATXN1[82Q] Purkinje neurons. Dendritic calcium spike threshold experiments were performed in both firing and non-firing cells. In non-firing ATXN1[82Q] Purkinje neurons, calcium spike measurements were performed, and subsequently the cells were exposed to 1 μM tetrodotoxin and 100 μM CdCl_2_. Non-firing cells whose membrane potential began the recording at a membrane potential more depolarized than -45 mV and fell to a membrane potential more hyperpolarized than -55 mV upon exposure to 1 μM tetrodotoxin and 100 μM CdCl_2_ were deemed to be usable for subsequent analysis, as prior work in ATXN1[82Q] Purkinje neurons has demonstrated that these cells are capable of firing action potential trains in response to injected current and are therefore likely not depolarized due to slicing injury [15]. Cells which were not firing and were more hyperpolarized than -45 mV or did not respond to 1 μM tetrodotoxin and 100 μM CdCl_2_ by hyperpolarization to below -55 mV were excluded, as these cells are typically unable to fire action potentials and are therefore likely a product of slicing injury.

Input resistance for each cell was calculated by generating an input-output curve for membrane potential vs. injected current. This input-output curve encompassed only membrane potential measurements between -80 mV and -75 mV in an effort to minimize the influence of active conductances on measurement of input resistance. This method is consistent with a previous study analyzing dendritic calcium spike threshold in response to injection of somatic current [28].

**Adeno-associated virus (AAV) transduction of Purkinje neurons**. Both AAV constructs used in this study (BK-AAV and GFP-AAV) have been described previously [15]. Stereotaxic administration of AAV2/5 or sham PBS injection was performed on twenty-eight day old mice as described previously [15]. Each animal received four injections (two per hemisphere), with a medial and lateral site on each hemisphere targeted to the deep cerebellar nuclei. The coordinates of the medial sites were -6.4 millimeters antero-posterior (from Bregma), ±1.3 millimeters medio-lateral (from Bregma), -1.9 millimeters dorsoventral (from skull surface). The coordinates of the lateral injection sites were -6.0 millimeters antero-posterior (from Bregma), ±2.0 millimeters medio-lateral (from Bregma), -2.2 millimeters dorsoventral (from skull surface). For each injection site, ∼5.0 × 10^9^ viral genomes (3 μl total volume) or an equal volume of PBS was delivered at an infusion rate of 0.5 μl/min using a 10 μl Hamilton syringe (BD Biosciences). The number of viral genomes delivered was within a range capable of producing widespread viral transduction with a minimal glial response [29].

**Phenotype analysis**. Motor coordination was evaluated using a rotarod. The study was powered to detect a 25% improvement in motor performance and estimated to require at least eight mice in each group. The groups were randomly allocated within cage and balanced with respect to sex, age, and number in each cohort. Approximately one week after virus injection or sham surgery, all mice were handled for four consecutive days. Once handling was complete, mice were trained for four consecutive days on the rotarod. The first three days of training were done on an accelerated protocol (4 to 40 rpm, at a rate of 0.12 rpm/s) and the last day was done at a constant speed of 24 rpm. Following the training period, mice were tested on the rotarod at a constant speed of 24 rpm on five consecutive days, with four trials per day. Time to task failure (reported as time to fail) was recorded as the time taken before the animal either fell off the bar or if an animal made three full rotations on the rotating rod, to a maximum time of 300 s. During both training and testing, injected mice were given twice-daily intraperitoneal injections of either baclofen (10 mg/kg in PBS) or an equal volume of PBS vehicle. After motor performance testing was complete, one cohort of mice used for rotarod testing was maintained on either baclofen-containing (150 mg/L) or vehicle drinking water until fourteen weeks of age, at which point mice were sacrificed and molecular layer thickness measurements were performed. A separate cohort of ATXN1[82Q] mice received sham surgery and received intraperitoneal injections of baclofen followed by baclofen-containing drinking water according to the schedule described above, and they were sacrificed at fourteen weeks of age for molecular layer thickness measurements. The tester remained blinded to genotype and treatment condition during experimentation. Performance on the rotarod was analyzed with a two-way repeated-measures ANOVA by trial with Holm-Sidak multiple comparison test.

**Tissue Immunohistochemistry**. Mice were anesthetized with isoflurane and brains were removed, fixed in 1% paraformaldehyde for 1 h, immersed in 30% sucrose in PBS and sectioned on a CM1850 cryostat (Leica). 14 μm parasagittal sections were processed for immunohistochemistry as described previously [15]. For molecular layer thickness measurements, Purkinje neurons were labelled with mouse anti-calbindin (1:1000, Cat. no. C9848, Sigma-Aldrich) and goat anti-mouse Alexa488 conjugated secondary antibody (1:200, Ref. no. A11001, Life Technologies Invitrogen). Sections were imaged using an Axioskop 2 plus microscope (Zeiss) at either 10X or 20X magnification. Measurements were performed using cellSens Standard image analysis software (Olympus). A line was drawn to measure 100 μm from the depth of the primary fissure along the length of the fissure. From the end of this line, a line was drawn to measure the distance to the nearest Purkinje neuron cell body in lobule V. Measurements of molecular layer thickness were performed in two sections per animal, and reported molecular layer thickness for each animal is the mean of these two measurements. Molecular layer thickness measurement was performed with experimenter blind to genotype and treatment condition.

**Chemicals**. Reagents and chemical were obtained from Sigma-Aldrich unless otherwise specified.

**Statistical analysis**. Statistical tests are described in the figure legends for all data. Data are expressed as mean ± SEM unless otherwise specified. Sample numbers are included in each panel, with the number of cells (n) or number of animals (N) included where appropriate. Studies were powered and analysis was performed assuming unequal variance between groups. Statistical significance for all Student’s T-test analysis was defined as *P<0*.*05*. For one-way ANOVA with Holm-Sidak multiple comparison test, adjusted *P<0*.*05* was considered statistically significant, and presented P values are adjusted P values. For two-way ANOVA, adjusted *P<0*.*05* was considered statistically significant, and presented P values are adjusted P values. For two-way repeated measures ANOVA, adjusted *P<0*.*05* was considered statistically significant, and presented P values are adjusted P values. For extra sum of squares F test, statistical significance was defined as a rejection of the null hypothesis that wild-type and ATXN1[82Q] data are better described by a single curve with *P<0*.*05*. Data were analyzed using SigmaPlot (Systat Software), GraphPad Prism (GraphPad), and Excel (Microsoft).

## Results

Qualitative changes in morphology of the dendrite have previously been reported to occur at the onset of motor dysfunction in the ATXN1[82Q] model of SCA1 [5]. To more quantitatively identify the time-course of dendritic degeneration in ATXN1[82Q] mice, we performed cell surface area estimation from Purkinje neurons in acute cerebellar slices using membrane capacitance, which is directly proportional to membrane surface area. The capacitative current generated during a voltage step was divided into a somatic/proximal dendritic component (C_1_) and a distal dendritic component (C_2_) using a well-established two-compartment equivalent circuit model of the Purkinje neuron [27]. Analysis of the distal dendritic component C_2_ revealed a reduction in distal dendritic surface area beginning at five weeks of age in ATXN1[82Q] mice, with a further progression of dendritic degeneration by fifteen weeks of age (Fig 1A). Measurement of the somatic and proximal dendritic contribution to cell capacitance (C_1_) revealed no changes to surface area of this compartment (Fig 1B). Together, these data establish that dendritic degeneration begins at five weeks of age in these mice, at the onset of motor impairment [5, 18].

Polyglutamine-expanded ATXN1, the disease-causing protein in SCA1, drives Purkinje neuron degeneration through its action in the nucleus [30] on regulators of transcription [31] and RNA processing [32]. Altered intrinsic excitability is known to drive Purkinje neuron degeneration in SCA1 mice [15, 18], suggesting that ion channel gene dysregulation may be an important effector pathway for SCA1 pathogenesis. We therefore explored a published RNA sequencing dataset from ATXN1[82Q] mice [24] (NCBI Gene Expression Omnibus accession number: GSE75778) to determine which members of the voltage-gated ion channel gene superfamily [25] are dysregulated in ATXN1[82Q] cerebella. Analysis of expression for all voltage-gated channel genes (see S1 and S2 Tables) at the onset of dendrite degeneration (Fig 1C) and in more advanced disease (Fig 1D) reveals altered expression of genes from multiple voltage-gated channel subfamilies. K^+^ channels, which make up the largest voltage-gated ion channel subfamily, are also the largest subfamily of dysregulated genes in this dataset. Increased Purkinje neuron intrinsic excitability is a feature of SCA1 [15], and this physiologic phenotype would be most parsimoniously explained by the observed reductions in K^+^ channel gene expression. We therefore focused our analysis on K^+^ channels and their potential role in dendritic degeneration.

Among the K^+^ channels downregulated in ATXN1[82Q] cerebella, many are known to be expressed [20, 33–36] and in some cases functionally important for Purkinje neuron dendrites [20, 21, 34]. Notably, *Kcnc3* (encoding K_v_3.3) and *Kcnma1* (encoding BK) are downregulated throughout disease and play important roles in limiting the excitability of Purkinje neuron dendrites [20, 21]. These data demonstrate that SCA1 Purkinje neurons show reduced expression of K^+^ channels, which would be predicted to increase dendritic membrane excitability.

**Fig 2:**
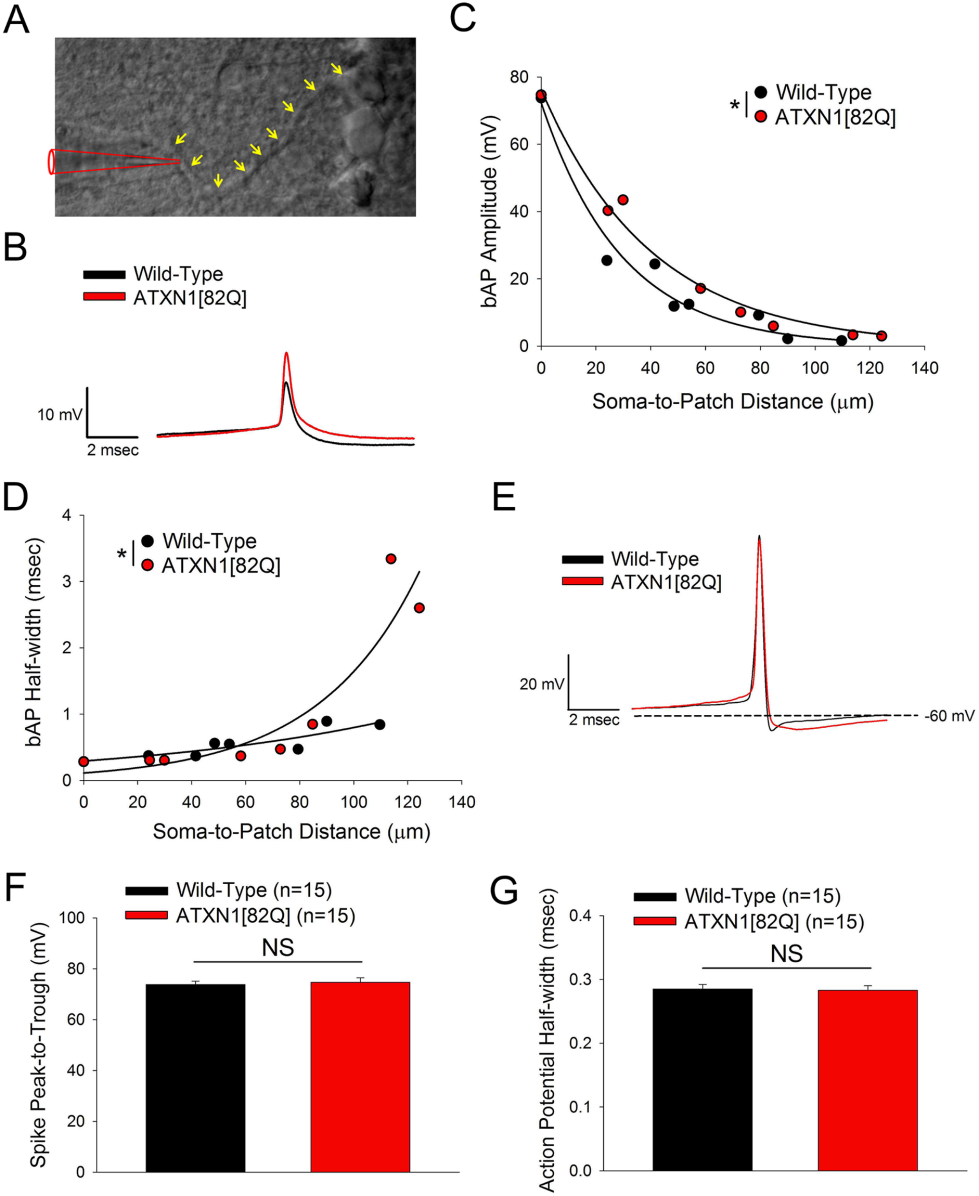
Increased Purkinje neuron dendritic membrane excitability is present at five weeks. **(A)** Representative image of dendritic patch clamp recording configuration with patch pipette (red) recording from a Purkinje neuron dendrite (yellow arrows). **(B)** Representative dendritic whole-cell patch clamp recordings from a five week old wild-type Purkinje neuron (soma-to-patch distance: 53.9 μm) and five week old ATXN1[82Q] Purkinje neuron (soma-to-patch distance: 58.2 μm). Traces have been aligned at the threshold of the spike. **(C)** Scatter-plots with single-exponent decay best-fit lines for back-propagating action potential (bAP) amplitude measurements in wild-type and ATXN1[82Q] Purkinje neurons at five weeks of age show less attenuation of back-propagating action potentials in ATXN1[82Q] mice. Each data point represents a recording from a separate cell. Best-fit line is an exponential decay model with two parameters, of the form *y* = *Ae*^−*bx*^, consistent with a previous study of action potential backpropagation in Purkinje neurons [37] and showing R^2^ values of 0.9717 (wild-type) and 0.9873 (ATXN1[82Q]). **(D)** Scatter-plots with single-exponent growth best-fit lines for bAP half-width measurements in wild-type and ATXN1[82Q] Purkinje neurons at five weeks of age show wider bAPs in ATXN1[82Q] mice. Each data point represents a recording from a separate cell. Best-fit line is an exponential growth model with two parameters, of the form *y* = *Ae*^*bx*^. **(E)** Representative action potential from a somatic whole-cell recording in wild-type and ATXN1[82Q] Purkinje neurons at five weeks of age. The peak-to-trough amplitude of action potentials (summarized in **(F)**) and the action potential half-width (summarized in **(G)**) recorded at the soma do not differ significantly between wild-type and ATXN1[82Q] Purkinje neurons at five weeks of age. In **(C**, **D**, **F**, and **G)** N = 5–10 mice were utilized per genotype. Where error bars are present, data are mean ± SEM. * = *P<0*.*05*; NS = Not significant. Statistical significance derived by extra sum of squares F test to determine whether one should reject the null hypothesis that data are best described by a single curve rather than two curves separated by genotype **(C**, **D)** or unpaired two-tailed Student’s T-Test **(F**, **G)**.

To explore how these changes in K^+^ channel expression impact ATXN1[82Q] Purkinje neuron dendritic excitability, we investigated action potential back-propagation at the onset of dendritic degeneration at five weeks. In addition to travelling down the axon, action potentials generated in the axon initial segment back-propagate into the dendritic tree, where they attenuate with distance from the soma [37] in a manner regulated by dendritic potassium channels [20–22]. A larger amplitude of back-propagating action potentials travelling into the dendritic tree represents an increase in dendritic membrane excitability. Direct patch-clamp recordings (Fig 2A) from dendrites were therefore used to look at the efficiency of back-propagation of action potentials. Wild-type Purkinje neurons show a robust attenuation of back-propagating action potentials travelling into the dendritic arbor, consistent with prior observations [20, 22, 37]. Patch-clamp recordings from the dendrite in five week old ATXN1[82Q] Purkinje neurons showed statistically-significantly less attenuation of back-propagating action potentials traveling into the dendritic tree than was observed for wild-type Purkinje neurons (Fig 2B and 2C). While modest, the effect is similar to pharmacologic inhibition of K^+^ channels that regulate Purkinje neuron dendritic excitability [20–22]. Furthermore, the best-fit lines for these data reveal a decay constant of 46.5 μm in ATXN1[82Q] Purkinje neurons and 26.6 μm in wild-type neurons (ATXN1[82Q]: *V*_*m*_ = 75.40*e*^−0.0215^, Wild-Type: *V*_*m*_ = 72.86*e*^−0.0376^), which would have a biologically-significant impact on dendritic physiology. Analysis of back-propagating action potential half-width revealed statistically significant widening in ATXN1[82Q] Purkinje neurons (Fig 2D), although this effect is unlikely to be biologically significant as it was limited to only a few distal recordings. Nevertheless, the increased back-propagating action potential amplitude suggests that there is a repolarization defect in the dendrite secondary to loss of one or more active K^+^ conductances. Increased action potential back-propagation has been observed with pharmacologic blockade either of K_v_3.3 or BK channels [20, 21], suggesting a role for both of these channels. The increased amplitude and width of back-propagating action potentials recorded in the dendrite was not explained by a change in amplitude or width of the action potential measured at the soma, which was similar in wild-type and ATXN1[82Q] Purkinje neurons (Fig 2E–2G). These results suggest that there is an increase in dendritic membrane excitability at the onset of dendritic degeneration.

**Fig 3:**
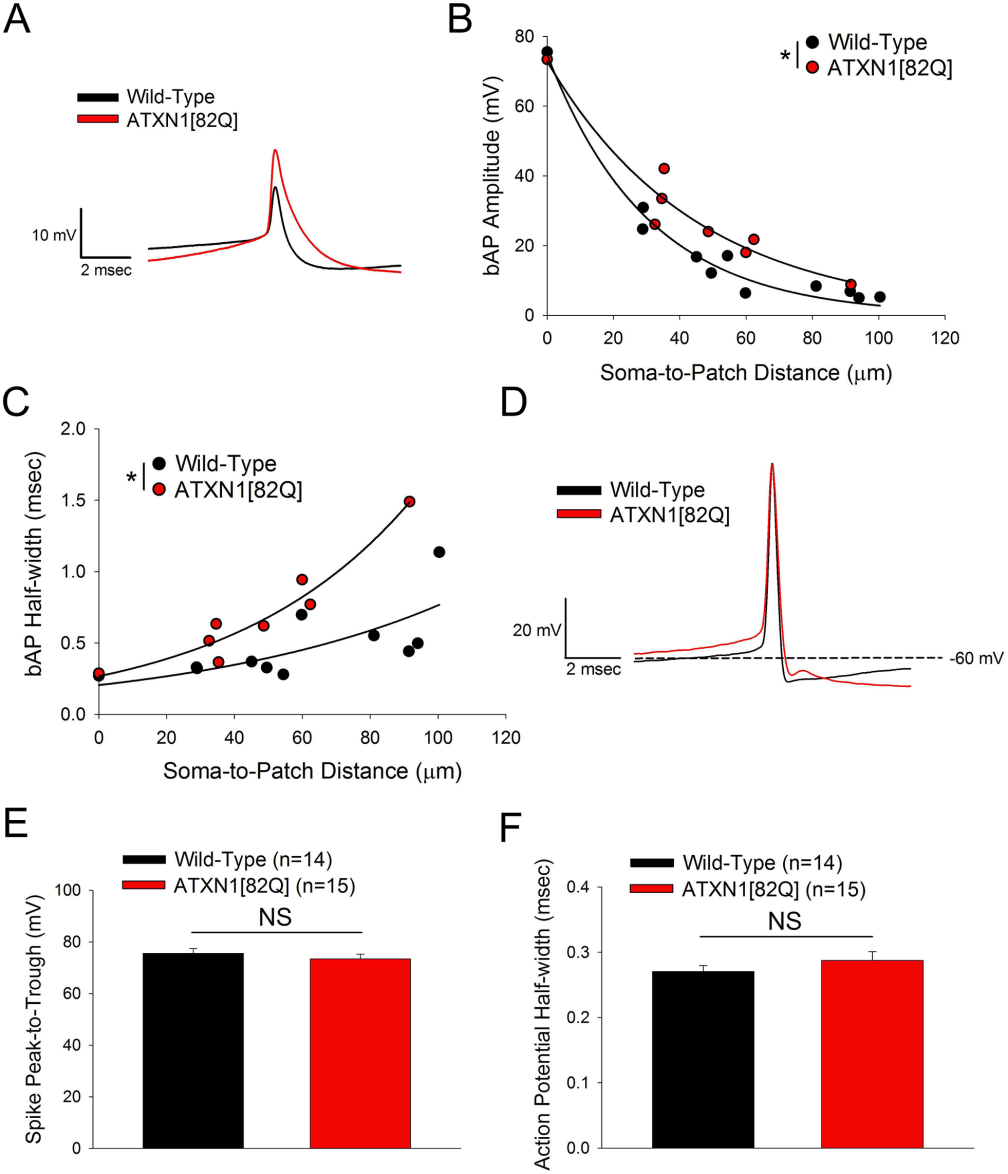
Increased dendritic membrane excitability persists in fifteen week atrophic SCA1 Purkinje neurons. **(A)** Representative dendritic whole-cell patch clamp recordings from a fifteen week old wild-type Purkinje neuron (soma-to-patch distance: 45.0 μm) and fifteen week old ATXN1[82Q] Purkinje neuron (soma-to-patch distance: 48.6 μm). Traces have been aligned at the threshold of the spike. **(B)** Scatter-plots with single-exponent decay best-fit lines for back-propagating action potential (bAP) amplitude measurements in wild-type and ATXN1[82Q] Purkinje neurons at fifteen weeks of age show less attenuation of bAPs in ATXN1[82Q] mice. Each data point represents a recording from a separate cell. Best-fit line is an exponential decay model with two parameters, of the form *y* = *Ae*^−*bx*^, consistent with a previous study of action potential backpropagation in Purkinje neurons [37] and showing R^2^ values of 0.9834 (wild-type) and 0.9346 (ATXN1[82Q]). **(C)** Scatter-plots with single-exponent growth best-fit lines for bAP half-width measurements in wild-type and ATXN1[82Q] Purkinje neurons at fifteen weeks of age show wider bAPs in ATXN1[82Q] mice. Each data point represents a recording from a separate cell. Best-fit line is an exponential growth model with two parameters, of the form *y* = *Ae*^*bx*^. **(D)** Representative action potential from a somatic whole-cell recording in wild-type and ATXN1[82Q] Purkinje neurons at fifteen weeks of age. The peak-to-trough amplitude of action potentials (summarized in **(E)**) and the action potential half-width (summarized in **(F)**) recorded at the soma do not differ significantly between wild-type and ATXN1[82Q] Purkinje neurons at fifteen weeks of age. In **(B**, **C**, **E**, and **F)** N = 5–10 mice were utilized per genotype. Where error bars are present, data are mean ± SEM. * = *P<0*.*05*; NS = Not significant. Statistical significance derived by extra sum of squares F test to determine whether one should reject the null hypothesis that data are best described by a single curve rather than two curves separated by genotype **(B**, **C)** or unpaired two-tailed Student’s T-Test **(E**, **F)**.

In the ATXN1[82Q] model of SCA1, progressive dendritic degeneration precedes overt cell loss. In a prior study, it was demonstrated that homeostatic changes in K^+^ channels resulting from cell atrophy at fifteen weeks helped to preserve spiking [15]. To assess whether cell atrophy plays a similar role in normalizing dendritic membrane excitability, we performed dendritic patch clamp recordings in ATXN1[82Q] Purkinje neurons at fifteen weeks of age. In wild-type Purkinje neurons at fifteen weeks there was robust attenuation of back-propagating action potentials travelling into the dendritic arbor (Fig 3A and 3B), similar to what was seen at five weeks (see Fig 2). Direct dendritic patch clamp recordings from fifteen week old ATXN1[82Q] Purkinje neurons demonstrated statistically-significantly less attenuation of back-propagating action potentials than wild-type Purkinje neurons (Fig 3B), with a decay constant of 43.5 μm for ATXN1[82Q] Purkinje neurons and 26.0 for wild-type neurons (ATXN1[82Q]: *V*_*m*_ = 72.99*e*^−0.0230^, Wild-Type: *V*_*m*_ = 75.64*e*^−0.0385^). Additionally, there was a robust widening of back-propagating action potentials at all locations in the dendrite (Fig 3C). These observations reflect a dysfunction specific to the dendrite, as they occur despite the fact that the amplitude and half-width of the action potential were similar between ATXN1[82Q] and wild-type Purkinje neurons at the soma (Fig 3D–3F). The more prominent widening of the back-propagating action potentials in ATXN1[82Q] Purkinje neurons relative to what was observed at five weeks of age (see Fig 2D) suggests an impact of progressive gene expression changes or involvement of additional K^+^ channels at fifteen weeks. Notably, reduced expression of *Kcnd3*, which encodes the A-type K^+^ channel K_v_4.3, is observed at twelve weeks but not five weeks of age (see Fig 1), and A-type K^+^ channels play a role in repolarization of back-propagating action potentials in CA1 pyramidal neurons [38]. Overall, these data suggest that the increase in dendritic membrane excitability persists at all stages of disease in ATXN1[82Q] Purkinje neurons.

**Fig 4:**
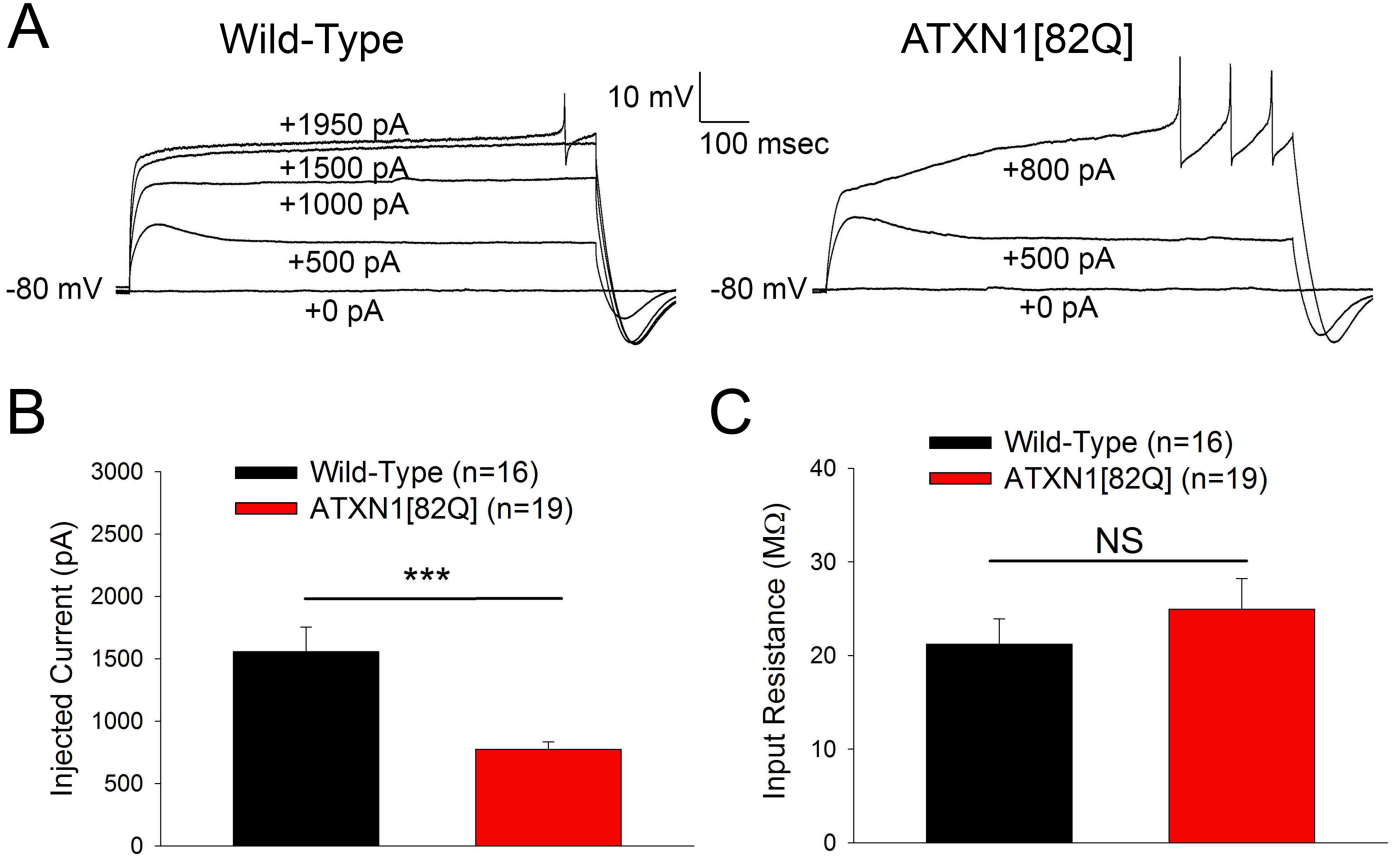
SCA1 mice show reduced threshold to evoke calcium spikes. **(A)** Representative traces where dendritic calcium spikes were evoked with somatic current injection (injected current amount indicated on the trace) from five week old wild-type Purkinje neurons (left) and ATXN1[82Q] Purkinje neurons (right) in the presence of 1 μM TTX. ATXN1[82Q] Purkinje neurons require less injected current to elicit a dendritic calcium spike than wild-type Purkinje neurons, summarized in **(B)**. **(C)** At five weeks of age, the input resistances of wild-type and ATXN1[82Q] Purkinje neurons do not differ when measured in normal aCSF with 1 μm TTX. In **(B** and **C)** N = 4–6 mice were utilized per genotype. Throughout, data are mean ± SEM. *** = *P<0*.*001*; NS = Not significant.

Purkinje neurons are similar to some other types of neurons in that they show locally-generated dendritic spikes, which for Purkinje neurons are mediated by voltage-gated calcium channels [39]. To measure the consequences of increased dendritic excitability for dendritic calcium spikes, we measured the threshold to evoke calcium spikes in response to somatic depolarization. Five week old ATXN1[82Q] Purkinje neurons required less injected current to elicit a dendritic calcium spike relative to age-matched wild-type controls (Fig 4A and 4B) without a change in input resistance (Fig 4C). Dendritic calcium spikes in Purkinje neurons have previously been shown to be regulated by BK channels [40] and K_v_3.3 channels [28]. These results suggest that reduced K^+^ channel expression in ATXN1[82Q] Purkinje neurons increases the propensity for regenerative calcium spikes in the dendrite.

**Fig 5:**
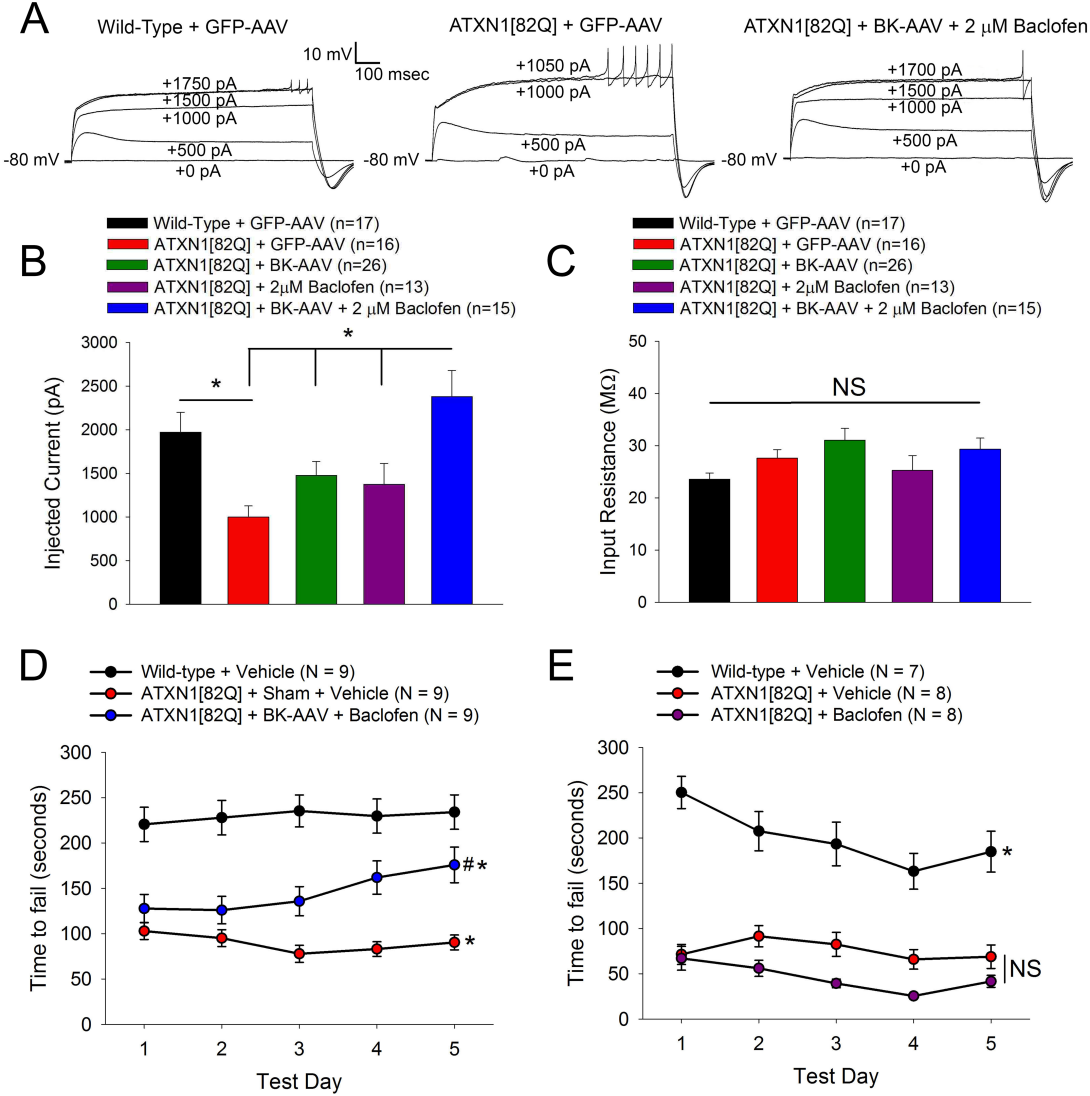
Rescuing intrinsic dendritic excitability improves SCA1 mouse motor performance. **(A)** Representative traces from Purkinje neurons at five weeks of age where dendritic calcium spikes were evoked with somatic current injection (injected current amount indicated on the trace). Recordings are from wild-type mice treated with GFP-AAV (left), ATXN1[82Q] mice treated with GFP-AAV (middle), and ATXN1[82Q] mice treated with BK-AAV and recorded in the presence of 2 μm baclofen (right). **(B)** At five weeks of age, Purkinje neurons from ATXN1[82Q] mice which had been injected with BK-AAV and recorded in the presence of 2 μm baclofen and 1 μM TTX show a normalization of their dendritic calcium spike threshold. Data are mean ± SEM. * = *P<0*.*05*. Statistical significance derived by one-way ANOVA with Holm-Sidak multiple comparison test with. N = 4–6 mice were utilized per group. **(C)** Input resistance is consistent across all conditions tested when measured in normal aCSF with 1 μm TTX. Data are mean ± SEM. * = *P<0*.*05*. Statistical significance derived by one-way ANOVA with Holm-Sidak multiple comparison test with. N = 4–6 mice were utilized per group. **(D)** Rotarod task performance was improved in ATXN1[82Q] mice injected with BK-AAV and treated with baclofen, when compared to ATXN1[82Q] mice that received sham surgery and were treated with vehicle. Wild-type mice that were treated with vehicle were used as a control. Data are mean ± SEM. * = statistically-significant difference from Wild-Type + Vehicle; #* = statistically-significant difference ATXN1[82Q] + Sham + Vehicle. Statistical significance derived by repeated-measures two-way ANOVA with Holm-Sidak multiple comparison test. **(E)** Rotarod task performance was not improved in ATXN1[82Q] mice treated with baclofen alone, when compared to ATXN1[82Q] mice that were treated with vehicle. Untreated wild-type mice were used as a control. Data are mean ± SEM. * = statistically-significant difference from ATXN1[82Q] + Vehicle and ATXN1[82Q] + Baclofen; NS = not significant.

To directly test a role for dendritic K^+^ channel dysfunction in disease pathogenesis, we aimed to first identify a treatment strategy which could normalize dendritic excitability in ATXN1[82Q] Purkinje neurons. We chose to target K^+^ channels, as our findings to this point suggested that reduced transcript levels for K^+^ channel found in the dendrite lead to dendritic hyperexcitability in ATXN1[82Q] mice. Our previous work in ATXN1[82Q] mice demonstrated reduced expression of large-conductance calcium-activated potassium (BK) channels [15],which are expressed in Purkinje neuron dendrites [33] and regulate Purkinje neuron dendritic excitability [21, 41]. Furthermore, another study in ATXN1[82Q] mice found that the GABA_B_ agonist baclofen can be used to lower Purkinje neuron dendritic excitability through potentiation of dendritic K^+^ channels, although the specific channel targets were not determined [42]. Accordingly, we aimed to combine rescue of BK channel expression with baclofen in SCA1 Purkinje neurons to determine whether this treatment strategy could fully rescue intrinsic dendritic excitability at five weeks of age (Fig 5A and 5B). Rescue of BK expression using an adeno-associated virus (AAV) to drive exogenous BK channel expression [15] or baclofen treatment each produced an increase in dendritic calcium spike threshold, but only treatment with a combination of BK-AAV and baclofen restored ATXN1[82Q] Purkinje neurons’ dendritic calcium spike threshold back to wild-type levels. Importantly, none of these changes in dendritic calcium spike threshold can be attributed to changes in cell input resistance (Fig 5C). These data suggest that treatment with both BK-AAV and baclofen is an effective strategy for normalizing dendritic excitability in ATXN1[82Q] Purkinje neurons.

To determine whether increased dendritic excitability plays a role in motor dysfunction in ATXN1[82Q] mice, we analyzed the effect of BK-AAV and baclofen treatment on rotarod performance. Motor impairment was assessed by rotarod performance two weeks after initiation of therapy, at which point combined treatment with BK-AAV and baclofen significantly improved motor performance of ATXN1[82Q] mice (Fig 5D). Rotarod testing in a separate cohort of ATXN1[82Q] mice treated with baclofen alone revealed that baclofen therapy did not improve motor performance (Fig 5E), and a previous study has demonstrated that BK-AAV treatment by itself produces no detectable improvement in motor performance at this time point [15], suggesting that combined treatment is required to observe improvement in motor dysfunction. BK-AAV treatment rescues spiking defects at this age [15] and baclofen treatment rescues mGluR signaling defects at this age [43], but neither treatment alone is able to rescue motor dysfunction at this age. This suggests that the activation of dendritic K^+^ channels mediates the improvement in motor impairment at this stage of disease in ATXN1[82Q] mice.

**Fig 6:**
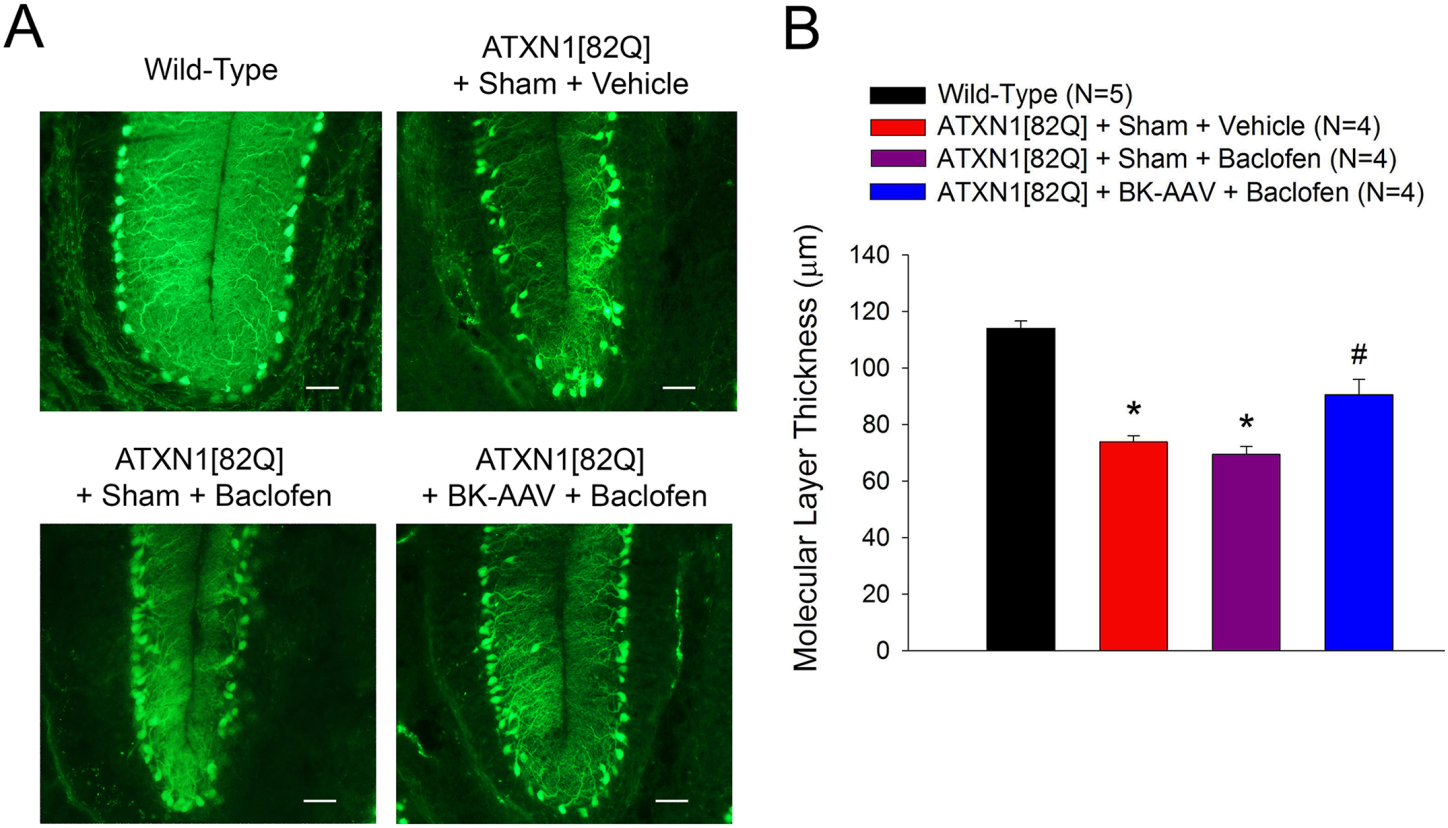
Normalizing intrinsic dendritic excitability limits SCA1 Purkinje neuron dendritic degeneration. **(A)** Representative images at the cerebellar primary fissure in wild-type mice, ATXN1[82Q] mice exposed to sham surgery and vehicle, ATXN1[82Q] mice exposed to sham surgery and baclofen, and ATXN1[82Q] mice exposed to BK-AAV and baclofen. Scale bar, 50 μm. **(B)** Molecular layer thinning is reduced at fourteen weeks in ATXN1[82Q] mice injected with BK-AAV and treated with baclofen. Data are mean ± SEM. * = statistically-significant difference from wild-type and ATXN1[82Q] BK-AAV + Baclofen groups; # = statistically-significant difference from all other groups. Statistical significance derived by one-way ANOVA with Holm-Sidak multiple comparison test.

To test the hypothesis that increased dendritic excitability contributes to dendritic degeneration in the ATXN1[82Q] model of SCA1, we examined the consequences of improving dendritic excitability on dendritic degeneration. Combined treatment with BK-AAV and baclofen limits the thinning of Purkinje neuron dendrites in the cerebellar molecular layer (Fig 6A and 6B). Previous studies have shown that BK-AAV treatment alone does not preserve molecular layer thickness despite being able to rescue somatic spiking [15], and in the present study baclofen alone does not preserve molecular layer thickness. Importantly, the fact that combined treatment with BK-AAV and baclofen can rescue dendritic excitability and limit degeneration of ATXN1[82Q] dendrites supports the conclusion that increased intrinsic dendritic excitability may contribute to dendritic degeneration in SCA1.

## Discussion

The current study demonstrates increased dendritic excitability associated with K^+^ channel dysfunction in SCA1, and also finds that a treatment strategy which improves dendritic excitability also slows dendritic degeneration. Changes in K^+^ channel function that result in altered spiking in Purkinje neurons [7, 13, 15, 17, 19, 44–47] and other neurons [48] are known to be associated with neurodegeneration. Prior work has suggested that degenerative cell atrophy may have a homeostatic function wherein increasing K^+^ channel density helps preserve Purkinje neuron pacemaking [15, 17]. The current work suggests that in the dendritic compartment similar homeostatic processes either do not exist or are unable to normalize membrane excitability, and that increased dendritic excitability persists at all stages of disease. This could explain how changes in excitability contribute to continuing neurodegeneration despite apparent homeostatic restoration of K^+^ channel function in spiking. Although the focus of this study was on the role for K^+^ channels in dendritic excitability, we noted alterations in mRNA transcripts of other ion channels that play a role in regulating dendrite excitability. For example, transcripts of the calcium channels, Cacna1c and Cacna1i are upregulated simultaneously with Cacna1g downregulation at 5 weeks. Dendritic excitability would be expected to be increased by increased expression of these calcium channels, and reduced due to their downregulation. It is important to note that in spite of persistently reduced Cacna1g expression at 15 weeks, however, dendritic excitability remains increased in parallel with reduced K^+^ channel transcripts. While there are other studies investigating intrinsic dendritic excitability in models of ataxia [42, 49], ours is the first to suggest a potential role for changes in intrinsic dendritic excitability as a driver of Purkinje neuron degeneration. Importantly, in this study we identify alterations in excitability that are correlated with reduced expression of specific potassium channels, and we find that a treatment strategy which targets dendritic K^+^ channels is able to reduce dendritic excitability and improve dendritic degeneration.

Several caveats should be taken into consideration in interpreting this study. The first is that the impact of BK-AAV and baclofen treatment is not limited to dendritic excitability, making it unclear to what extent normalization of dendritic excitability mediates the observed motor improvement and neuroprotection. Prior publications have rescued motor performance in SCA1 mice with BK-AAV [15] or baclofen [43], and in these studies it was proposed that the rescue of motor performance was through normalization of Purkinje neuron pacemaking and mGluR activation, respectively. Notably, we find a synergistic effect of BK-AAV and baclofen on improving dendritic degeneration (Fig 6A and 6B) that closely mirrors the synergistic effect of BK-AAV and baclofen on reducing dendritic excitability (Fig 5A and 5B), supporting our conclusion that BK-AAV and baclofen treatment could rescue dendritic degeneration through its impact on dendritic excitability. It is possible that the improvement in motor function and reduction in dendrite loss represents a non-specific improvement of Purkinje neuron health. Although we did not administer BK-AAV in combination with baclofen to wild-type mice in this study, administration of pharmacological activators of K^+^ channels improved motor function in a genotype specific manner in a prior study [42]. We cannot also exclude the possibility that our observation of motor improvement and neuroprotection with BK-AAV and baclofen occurs through effects on spiking, mGluR signaling, or other features of Purkinje neuron physiology impacted by this treatment strategy.

The second caveat to be considered is the fact that Purkinje neuron dendrites undergo structural changes throughout the course of disease, and changes in dendrite structure would be predicted by themselves to produce changes in waveform propagation through the dendritic arbor [50]. In the present study, treatment strategies which activated dendritic K^+^ channels could produce significant rescue in dendritic calcium spike propensity, implying that there is a significant role for K^+^ channel dysfunction in the physiologic phenotype of ATXN1[82Q] Purkinje neurons. Nevertheless, the relative contributions of dendrite remodeling vs. dendrite K^+^ channel dysfunction to the observed changes in dendritic physiology cannot be determined from the present study.

The observed changes in intrinsic dendritic excitability are likely to have a significant impact on dendritic integration of synaptic inputs onto SCA1 Purkinje neurons. Specifically, reduced dendritic K^+^ channel function would be predicted to produce enhanced summation [51], increased amplitude, and slower decay kinetics of AMPA EPSPs [52], enhanced supralinear integration observed during strong parallel fiber activation [41], and changes in the shape and propagation of climbing fiber-induced complex spikes [20, 53]. In fact, experiments in ATXN1[82Q] Purkinje neurons suggest possible changes in parallel fiber EPSPs and enhanced dendritic calcium entry during complex spikes [54] although it is important to note that this study did not quantify those differences.

The incomplete rescue of motor performance and dendrite degeneration with combined BK-AAV and baclofen treatment raises questions about the degree to which dendritic K^+^ channel dysfunction contributes to disease. Might greater rescue have been achieved by also targeting other K^+^ channels affected in disease? K_v_3.3 could be a promising target given that reduced expression of K_v_3.3 is observed at all stages of disease and that function-impairing mutations in K_v_3.3 cause Spinocerebellar ataxia type 13 [55]. Importantly, a recent study using pharmacological activators of K^+^ channels in SCA1 mice found that targeting dendritic excitability was important for producing sustained motor improvement, arguing for the importance of addressing increased dendritic excitability in ataxia [42].

The findings from this study are likely to have implications for many other forms of cerebellar ataxia. Spinocerebellar ataxia type 13 (SCA13) and Spinocerebellar ataxia type 19/22 (SCA19/22) are caused by function-impairing mutations in the genes encoding the potassium channels K_v_3.3 [55] and K_v_4.3 [56], respectively, and studies of these channels or their paralogs have demonstrated that they limit dendritic excitability [28, 57]. Focusing on intrinsic dendritic excitability in Purkinje neurons from models of these other ataxias may yield important insights, and these ataxias may similarly respond to agents that target dendritic excitability. Additionally, changes in Purkinje neuron membrane excitability have been identified in other models of ataxia where the primary mutation is not in an ion channel gene. Many of these studies have identified changes in Purkinje neuron spiking associated with altered function of potassium channels that are highly-expressed in the dendrite [16, 17, 19, 45], raising the possibility that similar increases in dendritic excitability may be observed in these models. It will be important to explore dendritic excitability in these models, as it may represent a common mechanism of dendrite pathology across many forms of cerebellar ataxia.

Alterations in intrinsic dendritic excitability and their impact on neurodegeneration may extend to other neurodegenerative diseases. Studies in noradrenergic neurons of the locus coeruleus [58] and neurons in the dorsal motor nucleus of the vagus [59], both of which degenerate in Parkinson disease, suggest that these neuronal populations show a unique pattern of dendritic excitability and oscillatory calcium entry that may explain their vulnerability to Parkinson disease pathology. In addition, several studies in rodent models of Alzheimer disease have demonstrated increased dendritic excitability in CA1 pyramidal neurons in Alzheimer disease models [60, 61]. To the best of our knowledge, our study is the first to demonstrate that alterations in dendritic physiology may drive neuropathology in a model of cerebellar ataxia. These data present an exciting possibility: that targeting dendritic excitability represents a novel therapeutic strategy in ataxias and other neurodegenerative diseases.

## Supporting information

S1 Table Cerebellar gene expression for all voltage-gated ion channel genes in five week old ATXN1[82Q] mice. Data table comparing expression of all voltage-gated ion channel genes with official IUPHAR classification [25] in ATXN1[82Q] cerebellar samples relative to FVB wild-type cerebellar samples at five weeks of age. The data table reflects a subset of previously-published RNA sequencing analysis of gene expression in ATXN1[82Q] mice [24], and contains fold change expression (ATXN1[82Q] relative to wild-type), p-value, and q-value generated as part of this prior analysis. Those ion channel genes where differential expression showed q≤0.05 were defined as statistically-significantly altered in their expression, consistent with the original analysis [24], and the gene names are marked with (**).
(XLSX)

S2 Table Cerebellar gene expression for all voltage-gated ion channel genes in twelve week old ATXN1[82Q] mice. Data table comparing expression of all voltage-gated ion channel genes with official IUPHAR classification [25] in ATXN1[82Q] cerebellar samples relative to FVB wild-type cerebellar samples at twelve weeks of age. The data table reflects a subset of previously-published RNA sequencing analysis of gene expression in ATXN1[82Q] mice [24], and contains fold change expression (ATXN1[82Q] relative to wild-type), p-value, and q-value generated as part of this prior analysis. Those ion channel genes where differential expression showed q≤0.05 were defined as statistically-significantly altered in their expression, consistent with the original analysis [24], and the gene names are marked with (**).
(XLSX)
